# Comparison of quantitative radiomorphometric predictors of healthy and MRONJ-affected bone using panoramic radiography and cone-beam CT

**DOI:** 10.1093/dmfr/twae024

**Published:** 2024-05-29

**Authors:** Elif Aslan, Erinc Onem, Ali Mert, B Guniz Baksi

**Affiliations:** Department of Oral and Maxillofacial Radiology, Ege University School of Dentistry, Izmir, 35040, Turkey; Department of Oral and Maxillofacial Radiology, Ege University School of Dentistry, Izmir, 35040, Turkey; Department of Statistics, Ege University School of Science, Izmir, 35040, Turkey; Department of Oral and Maxillofacial Radiology, Ege University School of Dentistry, Izmir, 35040, Turkey

**Keywords:** cone-beam CT, medication-related osteonecrosis of the jaws, panoramic radiography, quantitative bone parameters

## Abstract

**Objectives:**

To determine the most distinctive quantitative radiomorphometric parameter(s) for the detection of MRONJ-affected bone changes in panoramic radiography (PR) and cone-beam CT (CBCT).

**Methods:**

PR and sagittal CBCT slices of 24 MRONJ patients and 22 healthy controls were used for the measurements of mandibular cortical thickness (MCT), fractal dimension (FD), lacunarity, mean gray value (MGV), bone area fraction (BA/TA), trabecular thickness (Tb.Th), trabecular separation (Tb.Sp), trabecular number (Tb.N). MCT was measured in the mental foramen region. While FD and lacunarity were measured on mandibular trabecular and cortical regions-of-interest (ROIs), the remaining parameters were measured on trabecular ROIs. The independent samples *t*-test was used to compare the measurements between the MRONJ and control groups for both imaging modalities (*P* = .05).

**Results:**

MCT was the only parameter that differentiated MRONJ-affected bone in both PR and CBCT (*P* < .05). None of the remaining parameters revealed any difference for MRONJ-affected bone in CBCT (*P* > .05). FD, lacunarity, MGV, BA/TA, and Tb.Sp could distinguish MRONJ-affected trabecular bone in PR (*P* < .05). The correspondent ROI for both imaging methods that was reliable for detecting MRONJ-affected bone was the trabecular bone distal to the mental foramen above the inferior alveolar canal (ROI-3).

**Conclusions:**

MCT is a reliable parameter for the discrimination of MRONJ-affected bone in both PR and CBCT images. PR may be used to detect MRONJ-affected trabecular bone using FD, lacunarity, MGV, BA/TA, and Tb.Sp measurements as well.

## Introduction

Medication-related osteonecrosis of the jaws (MRONJ) is the progressive destruction of alveolar bones caused by the adverse effects of anti-resorptive and anti-angiogenic drugs used to treat osteometabolic and/or oncologic disorders.^1^ Although many new drug groups have been identified, more than 90% of MRONJ cases have been reported to occur as a result of the use of IV bisphosphonates or subcutaneous denosumab-so called anti-resorptive therapy (ART).^2^

Patients with MRONJ are classified into different stages based on the severity of the pathology, and the treatment approach varies accordingly. While the recommended treatment protocol includes the improvement of oral hygiene and conservative treatment(s) for early stages, invasive surgical procedures may become necessary for advanced-stage patients.^1^ Nevertheless, the prognosis and compliance with the treatment decrease in advanced-stages due to widespread necrotic bone areas, severe inflammation, and pathological fractures.^2^ In order to ensure early diagnosis and prevent MRONJ-related complications, the American Society for Bone and Mineral Research (ASBMR) have recommended the use of non-invasive diagnostic and radiographic methods to delineate the early signs and predictors of MRONJ.^3^ It has been clearly defined that clinical findings of MRONJ may be insufficient and misleading for diagnosis and therefore imaging methods have become the first choice for the detection of bone alterations, particularly in patients with no exposed necrotic bone.^1^^,^^4^ However, unreliable, non-specific, and observer-dependent nature of qualitative radiographic findings may lead to under- or overestimation of the pathology, and therefore the use of quantitative radiomorphometric parameters becomes imperative for the proper identification of MRONJ-affected bone alterations.^5^^,^^6^

Quantitative bone measurements performed on radiographic images are objective methods determined by mathematical calculations and can provide adequate information for the detection of early structural bone changes before the bone destruction becomes visible on radiographs.^7^^,^^8^ Numerous studies on osteoporosis patients have proven that early bone degeneration can be detected by the measurement of quantitative bone parameters using two-dimensional (2D) and three-dimensional (3D) images.^9–11^ Morphometric bone measurements such as trabecular thickness (Tb.Th), trabecular separation (Tb.Sp), and trabecular number (Tb.N) are well-proven bone parameters used to detect early bone changes of osteoporosis.^12^^,^^13^ Moreover, recent studies have proven that lacunarity and bone area fraction (BA/TA) can also provide sufficient information about the quality of osteoporotic bone.^11^^,^^14^^,^^15^

It has been revealed that the use of 3D images gives more reliable results in the measurement of morphometric parameters. However, previous micro-CT studies have also proved that bone measurements performed on 3D micro-CT volumes and 2D micro-CT slices have a positive correlation, which confirms that these parameters can be reliably measured in 2D images as well.^16^^,^^17^ Furthermore, Diba et al^11^ reported that BA/TA and Tb.Th measurements in periapical images of osteoporotic patients are positively correlated with femoral bone mineral density (BMD), verifying the efficiency of these bone parameters in 2D radiographic images.

MRONJ-related alterations of the jaw bones have been previously evaluated by the measurements of mandibular cortical thickness (MCT),^5^^,^^8^^,^^18–20^ fractal dimension (FD),^20–23^ and mean gray value (MGV)^18^^,^^19^^,^^24^ on either panoramic radiography (PR), CT, or cone-beam CT (CBCT) images. However, to our knowledge, the measurement of lacunarity, BA/TA, Tb.Th, Tb.Sp, and Tb.N has not been investigated in any other study for the detection of changes in MRONJ-affected bone. Furthermore, none of the studies have compared the reliability of the measurements of quantitative bone parameters in 2D and 3D dental images for the detection of changes in MRONJ-affected alveolar bone. In fact, there is no accepted protocol regarding the choice of imaging method for the detection of distinctive radiographic findings of MRONJ-affected bone as well as the most relevant quantitative bone parameter(s) pertinent to the related imaging modalities.

Therefore, the aim of this study was to compare the quantitative bone parameters of MRONJ patients and healthy individuals using PR and CBCT images and to determine the most relevant morphometric parameter(s) for the detection of changes in MRONJ-affected bone.

## Methods

### Ethical approval

The present study design was approved by the Institutional Ethical Committee (17.05.2022/22-5T/5) of the University and followed the principles of the Declaration of Helsinki.

### Selection of MRONJ patients and healthy controls

Anamnesis data of patients referred to the Department of Oral and Maxillofacial Radiology between 2018 and 2020 were examined retrospectively in the MetaSoft patient database (Cortex Biophysik GmbH, Leipzig, Germany) using the keywords bisphosphonate, cancer, osteoporosis, and osteonecrosis. Thousand patients containing at least one of these keywords were selected and re-evaluated. Patients using anti-resorptive and/or anti-angiogenic drugs but not clear about the drug type, and patients with a history of radiotherapy to the head and neck region and/or having metastases to the jaw bones were excluded. Twenty-four patients (16 female, eight male, mean age: 68.12 ± 11.2) among the remaining 278 who had been using anti-resorptive/anti-angiogenic drugs with histopathologically confirmed MRONJ in the mandibular posterior region(s) and had both PR and CBCT images in the image archive were selected and included to the study.

While CBCT volumes of either the right or left mandible were available for 16 patients, entire mandible volume was available for the remaining 8 patients. Therefore, 32 hemi-mandibles of 24 MRONJ patients were used for the trabecular bone measurements. Unfortunately, mandibular cortical bone was not completely imaged in some CBCT volumes. Hence, cortical bone measurements were done only on 26 hemi-mandibles.

In order to select the control group, PR and CBCT images of healthy individuals obtained within the same 6-month period for various reasons between 2018 and 2020 were evaluated retrospectively. Twenty-two individuals (14 female, eight male, mean age: 57.95 ± 8.27) without any osteometabolic disease and/or history of drug use affecting the jaw bones and without any intra-osseous lesions were selected and included to the control group. Similar to the MRONJ group, 34 hemi-mandibles and 28 hemi-mandibles of 22 controls were used for trabecular and cortical bone measurements, respectively.

### Image acquisition

Panoramic images of the MRONJ and control groups had been previously obtained with Kodak 8000 digital PR device (Kodak Carestream Health Inc., Trophy, France) using 68 kV, 8 mA, and 13.9 s exposure parameters. Similarly, CBCT images had been previously obtained using the Kodak 9000 3D (Kodak Carestream Health, Trophy, France) device with exposure parameters of 70 kV, 10 mA, 10.8 s, and 76 µm (50 × 37 mm FOV) or 200 µm (85 × 37 mm FOV) isotropic voxel size.

### ROI selection

Mental foramen was taken as the reference for the determination of trabecular and cortical regions-of-interest (ROIs) in both PR and CBCT images. Since mandibular posterior regions were used for the measurements in PR images, sagittal CBCT sections were preferred to ensure that exactly the same regions were measured in both image types.

**Figure 1.**
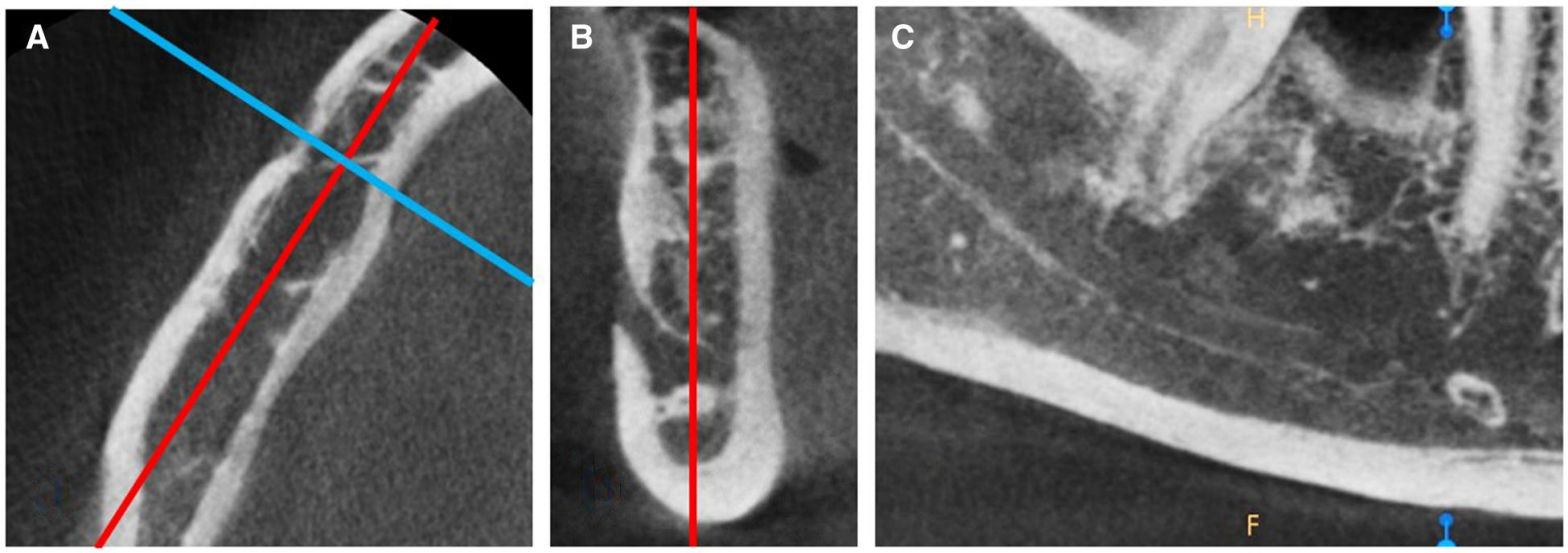
(A) The bucco-lingual line connecting the outer borders of buccal and lingual cortical walls of the mandible and passing through the centre of the mental foramen on axial slice (horizontal line). (A, B) The sagittal plane passing through the midpoint of the buccal and lingual cortical plates of the mandible on axial and coronal slices (vertical line). (C) The resulting sagittal plane for locating the ROI(s).

For the measurements in CBCT, first, axial and coronal slices in which the mental foramen was the widest were determined. Then, in order to create a bucco-lingual plane, a bucco-lingual line was drawn connecting the outer borders of buccal and lingual cortical walls of the mandible and passing through the centre of the mental foramen on the selected axial slice. Next, the sagittal plane passing through the midpoint of the bucco-lingual plane was selected to locate the ROI(s) for the measurements (Figure 1).

ROI sizes were determined by selecting the largest possible areas on both trabecular and cortical bone regions that would be equivalent in both PR and CBCT images. Care was taken to ensure that selected ROIs did not include any anatomical structures such as lamina dura of adjacent teeth, borders of inferior alveolar canal, and/or periapical regions that may produce misleading results.

**Figure 2.**
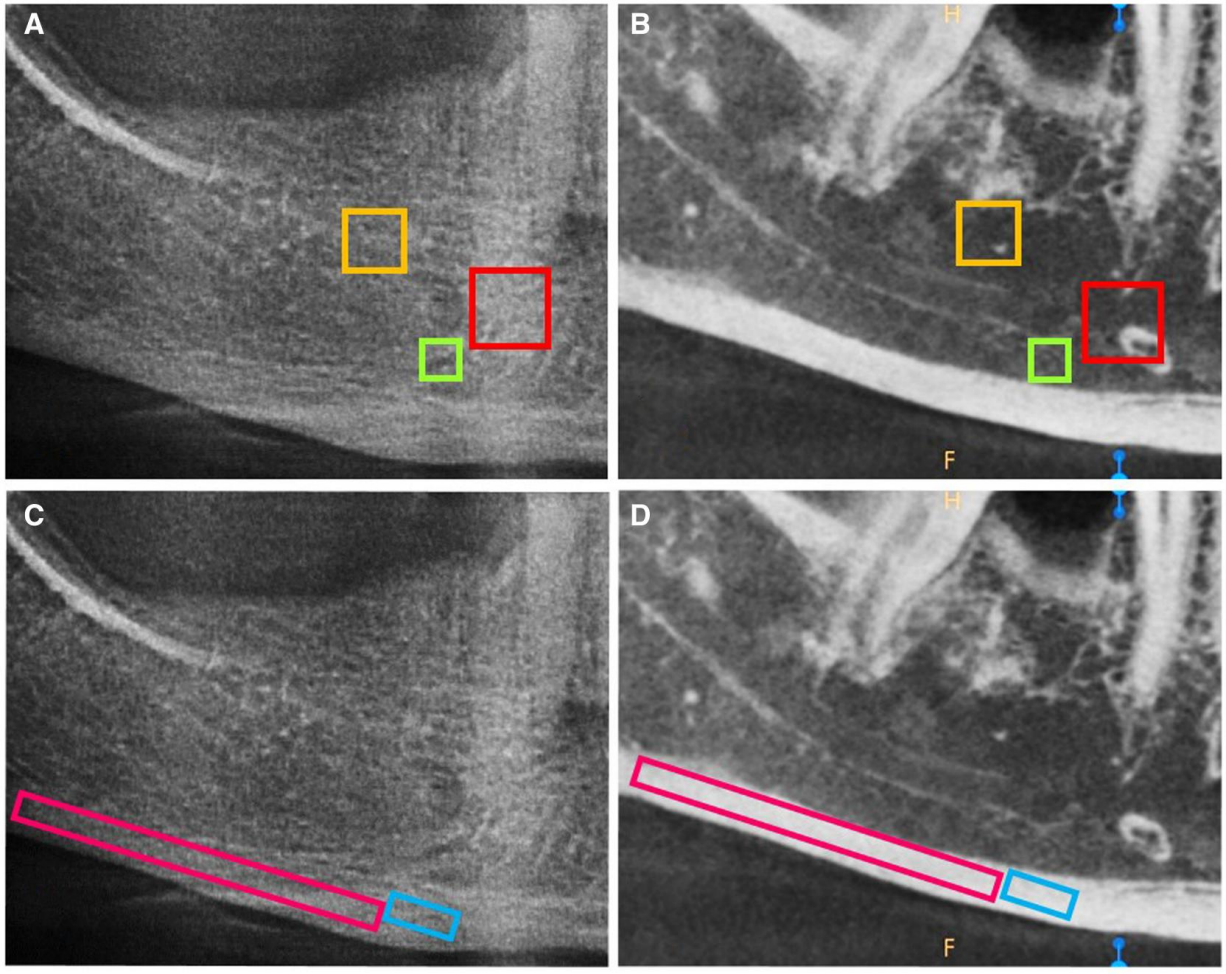
Locations of trabecular and cortical ROIs in PR and sagittal CBCT images. (A) PR and (B) CBCT images showing trabecular ROIs anterior to the mental foramen (ROI-1), inferior to the mental foramen (ROI-2), and distal to the mental foramen above the inferior alveolar canal (ROI-3). (C) PR and (D) CBCT images showing cortical ROIs inferior to the mental foramen (ROI-4), and between the distal border of the mental foramen to the ascending ramus (ROI-5).

To determine whether measurements would be affected by different ROIs of alveolar bone, three square trabecular and two rectangular cortical ROIs were selected and used for the measurements in both PR and CBCT images (Figure 2):

ROI-1: 50 × 50 pixels in the trabecular bone anterior to the mental foramen (Figure 2A-B),

ROI-2: 30 × 30 pixels in the trabecular bone inferior to the mental foramen (Figure 2A-B),

ROI-3: 40 × 40 pixels in the trabecular bone superior to the upper border of the inferior alveolar canal and distal to the mental foramen (Figure 2A-B),

ROI-4: 50 × 25 pixels, in the cortical bone inferior to the mental foramen and centered on the perpendicular line drawn from the centre of the mental foramen to the inferior base of the mandible (Figure 2C-D),

ROI-5: 350 × 25 pixels in the cortical bone extending from the distal border of the mental foramen to the ascending ramus (Figure 2C-D).

### Measurements

**Figure 3.**
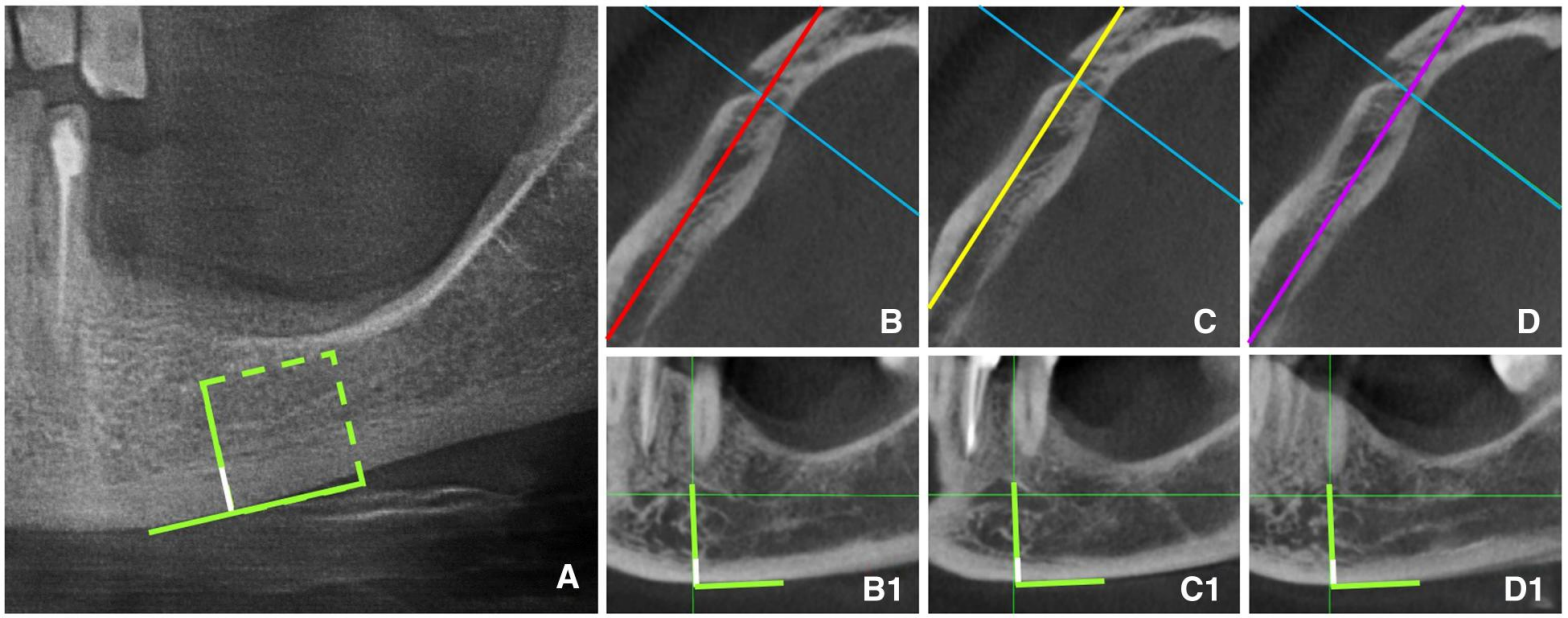
Measurement of MCT in PR and three sagittal CBCT slices. (A) PR image of the hemi-mandible showing the perpendicular line drawn to the tangent line to the inferior border. (B, B1) Measurement of MCT in the sagittal CBCT slice passing through the midpoint of the buccal and lingual cortical plates of the mandible, and the sagittal slices. (C, C1) 2 mm to the buccal, and (D, D1) 2 mm to the lingual to the middle slice. MCT was measured in the inferior part of the perpendicular line (white lines) for PR and each CBCT slice.

MCT measurements in PR and CBCT images were performed according to the method recommended by Taguchi et al^25^ For the measurements of MCT in both PR and CBCT images, a tangential line was drawn parallel to the inferior border of the mandible, and a perpendicular line was drawn to this tangent from the centre of the mental foramen. The inferior part of the perpendicular line covering the cortical bone was used for the cortical thickness measurement (Figure 3). Since cortical bone thickness may vary in different sagittal slices, MCT measurements were performed in three consecutive sagittal slices of CBCT images. The first sagittal slice was the slice described above, that was selected to locate the ROIs (Figure 3B-B1). Sagittal slices 2 mm to the buccal and lingual to this middle slice were used to measure MCT as well (Figure 3C-C1 and D-D1). The mean of three measurements was calculated as the CBCT value for the MCT of the relevant hemi-mandible.

For the measurements of other bone parameters (FD, lacunarity, MGV, BA/TA, Tb.Th, Tb.Sp, and Tb.N), trabecular and cortical ROIs of both PR and CBCT were segmented to obtain binary images by using Fiji software (ImageJ, National Institutes of Health, Bethesda, Maryland, ABD). Since Fiji software can only analyse foreground black pixels in a binary image, FD, BA/TA, MGV, and Tb.Th were calculated using binary images where bone areas were represented by black pixels. On the contrary, binary images where black pixels represented bone marrow were used to measure lacunarity and Tb.Sp.^26^

FD and lacunarity were measured on both trabecular (FD_tb,_ L_tb_) and cortical (FD_ct_, L_ct_) ROIs based on the previously proven fact that FD and lacunarity can significantly detect the alterations in both trabecular and cortical bone of osteoporosis patients.^9^^,^^10^ Since MGV, BA/TA, Tb.Th, Tb.Sp, and Tb.N are trabecular bone parameters and can only distinguish the alterations in trabecular bone, those were measured only on trabecular ROIs.

FD calculations on trabecular and cortical ROIs were performed using the *box counting* method.^27^ Lacunarity was calculated using *FracLac* plugin of the Fiji software.^28^

*Mean Gray Value* plugin was used for the calculation of MGV on trabecular ROIs by dividing the sum of the gray values of all pixels to the total number of pixels in the relevant ROI.^29^

BA/TA of trabecular ROIs was measured using the *Area Fraction* plugin—a plugin that calculates the percentage of pixels representing trabecular bone within the total bone area.^29^

**Figure 4.**
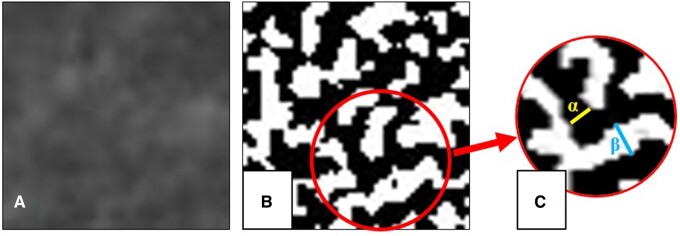
(A) Trabecular ROI cropped from the original image before binarization, (B) segmented binarized ROI showing bone trabeculae (black pixels) and bone marrow (white pixels), (C) graphical illustration of segmented image describing Tb.Th (α) and Tb.Sp (β).

Tb.Th and Tb.Sp were measured using the *Slice Geometry* feature of *BoneJ* plugin in Fiji for both PR and CBCT ROIs (Figure 4).^11^ Since there is no direct calculation method for Tb.N in 2D images using Fiji software, Tb.N was calculated using the following formula suggested by Steines et al: (Tb. Area × Tb. Thickness)/Total Area.^16^

### Statistical analysis

Data analysis was performed using the IBM SPSS Statistics 20.0 (SPSS Inc., Chicago, IL, United States). The mean and standard deviations of all measurements were calculated for MCT and for each ROI in PR and CBCT images of both the MRONJ and control groups.

In order to include the analysis of trabecular and cortical bone areas with variable osteoblastic and osteoclastic activity, the cumulative means of trabecular and cortical ROIs were calculated by taking the mean values of three trabecular (ROI-1, ROI-2, ROI-3) and two cortical ROIs (ROI-4, ROI-5) measured in both imaging methods.

The independent samples *t*-test was used to compare the differences between the cumulative means, measurements of different ROIs, and MCT measurements obtained in PR and CBCT images of the MRONJ and control groups. Significance level was determined as *P* = .05 for all comparisons.

All measurements were performed by a single oral and maxillofacial radiologist (EA) in order to minimize measurement errors that may arise from faulty ROI selection by different observers. For intra-observer agreement, measurements of 10 randomly selected patients from both the groups (30% of each group) were repeated in both PR and CBCT images. The reliability of the quantitative measurements was determined using Cronbach’s alpha analysis.^30^

## Results

The age, gender, diagnosis, drug type, and route of administration of MRONJ patients included to the study are presented in Table 1.

**Table 1.** Demographic and medical characteristics of MRONJ patients.

Number	Age	Gender	Diagnosis	Drug type	Route of administration
1	43	F	Breast cancer	Zoledronate	IV
2	85	F	Breast cancer	Bisphosphonate	IV
3	72	F	Osteoporosis	Alendronate	IV
4	57	F	Osteoporosis	Bisphosphonate	IV
5	78	F	Breast cancer	Bisphosphonate	IV
6	66	M	Osteoporosis	Ibandronate	IV
7	51	F	Osteoporosis	Bisphosphonate	IV
8	78	M	Prostate cancer	Zoledronate	IV
9	83	M	Prostate cancer	Zoledronate	IV
10	79	F	Osteoporosis	Bisphosphonate	IV
11	68	F	Multiple myeloma	Zoledronate	IV
12	66	M	Unspecified	Bisphosphonate	IV
13	64	M	Prostate cancer	Zoledronate	IV
14	55	F	Osteoporosis	Bisphosphonate	IV
15	57	F	Breast cancer	Zoledronate	IV
16	59	F	Multiple myeloma	Zoledronate	IV
17	67	M	Prostate cancer	Zoledronate	IV
18	84	F	Osteoporosis	Bisphosphonate	IV
19	69	M	Prostate cancer	Zoledronate	IV
20	67	F	Breast cancer	Zoledronate	IV
21	73	F	Unspecified	Bisphosphonate	IV
22	72	F	Breast cancer	Bisphosphonate	IV
23	83	M	Osteoporosis	Alendronate	IV
24	59	F	Lung cancer	Zoledronate	IV

Abbreviation: IV = intravenous.

The comparison of MCT measurements of the MRONJ and control groups disclosed that MCT obtained from MRONJ patients were significantly higher than healthy controls in both PR (*P* = .003) and CBCT images (*P* < .0001) (Table 2).

**Table 2.** Comparison of cumulative means of different ROIs measured in PR and CBCT of the MRONJ and control groups.

Imaging method	Parameter	MRONJ group Mean ± SD	Control group Mean ± SD	*P-*value^a^
PR	MCT	5.35 ± 1.04	4.64 ± 0.66	**.003** ^b^
	FD_tb_	1.46 ± 0.04	1.48 ± 0.02	**.017** ^b^
	FD_ct_	1.48 ± 0.03	1.49 ± 0.02	.141
	L_tb_	0.33 ± 0.04	0.3 ± 0.002	**.008** ^b^
	L_ct_	0.27 ± 0.03	0.27 ± 0.03	.515
	MGV	110.03 ± 9.47	115.81 ± 4.33	**.003** ^b^
	BA/TA	43.12 ± 3.74	45.40 ± 1.69	**.002** ^b^
	Tb.Th	4.06 ± 0.44	4.03 ± 0.2	.733
	Tb.Sp	4.99 ± 0.88	4.6 ± 0.39	**.02** ^b^
	Tb.N	1.73 ± 0.23	1.80 ± 0.09	.099
	MCT	5.78 ± 1.2	4.2 ± 0.54	**.000** ^b^
	FD_tb_	1.4 ± 0.04	1.41 ± 0.03	.241
	FD_ct_	1.4 ± 0.02	1.41 ± 0.03	.826
	L_tb_	0.37 ± 0.05	0.35 ± 0.03	.114
CBCT	L_ct_	0.35 ± 0.04	0.33 ± 0.04	.23
	MGV	107.01 ± 10.46	106.87 ± 6.02	.946
	BA/TA	42.33 ± 3.24	41.97 ± 2.38	.61
	Tb.Th	5.1 ± 0.52	4.9 ± 0.48	.12
	Tb.Sp	6.44 ± 0.88	6.28 ± 0.63	.401
	Tb.N	2.13 ± 0.29	2.04 ± 0.28	.173

Abbreviations: BA/TA = bone area fraction, CBCT = cone-beam CT, FD_ct_ = cortical fractal dimension, FD_tb_ = trabecular fractal dimension, L_c_ = cortical lacunarity, L_tb_ = trabecular lacunarity, MCT = mandibular cortical thickness, MGV = mean gray value, MRONJ = medication-related osteonecrosis of the jaws, PR = panoramic radiography, Tb.N = trabecular number, Tb.Sp = trabecular separation, Tb.Th = trabecular thickness.

Bold values indicate statistically significant.

a Independent samples *t*-test.

b *P* < .05.

The cumulative mean FD_tb_ (*P* = .017), MGV (*P* = .003), and BA/TA (*P* = .002) of the MRONJ group were significantly lower than the control group, whereas L_tb_ (*P* = .008) and Tb.Sp (*P* = .02) were significantly higher in PR images. No significant differences were obtained between the MRONJ and control groups regarding the cumulative means of FD_ct_, L_ct_, Tb.Th, and Tb.N measured in PR images (*P* > .05).

None of the parameters revealed any significant difference for MRONJ-affected bone in CBCT measurements (*P* > .05) (Table 2).

### Comparison of different ROIs

Comparison of measurements of different ROIs between the MRONJ and control groups demonstrated that panoramic measurements of FD_tb_ for the MRONJ group were lower than that of the control group on ROI-1 (*P* = .015) and ROI-3 (*P* = .007). On the other hand, L_tb_ and Tb.Sp were higher on ROI-1 (*P*_lac_ = .002, *P*_tb.sp_ = .018) and ROI-3 (*P*_lac_ = .048, *P*_tb.sp_ = .035) for the MRONJ group as compared to the measurements of the healthy controls (Table 3).

**Table 3.** Measurements of different ROIs in PR for the MRONJ and control groups.

Parameter	MRONJ Mean ± SD	Control Mean ± SD	*P*-value^a^
**FD_tb_**			
ROI-1	1.56 ± 0.04	1.58 ± 0.03	**.015** ^b^
ROI-2	1.42 ± 0.05	1.44 ± 0.03	.163
ROI-3	1.41 ± 0.04	1.44 ± 0.02	**.007** ^b^
**FD_ct_**			
ROI-4	1.45 ± 0.04	1.46 ± 0.03	.148
ROI-5	1.5 ± 0.03	1.51 ± 0.02	.23
**L_tb_**			
ROI-1	0.3 ± 0.05	0.27 ± 0.02	**.002** ^b^
ROI-2	0.36 ± 0.06	0.34 ± 0.04	.062
ROI-3	0.32 ± 0.04	0.3 ± 0.03	**.048** ^b^
**L_ct_**			
ROI-4	0.29 ± 0.03	0.29 ± 0.04	.963
ROI-5	0.25 ± 0.03	0.24 ± 0.03	.24
**MGV**			
ROI-1	110.35 ± 9.51	116.02 ± 4.47	**.004** ^b^
ROI-2	109.37 ± 10.8	115.44 ± 6.08	**.007** ^b^
ROI-3	110.36 ± 10.45	115.98 ± 4.52	**.008** ^b^
**BA/TA**			
ROI-1	43.28 ± 3.73	45.5 ± 1.75	**.004** ^b^
ROI-2	42.89 ± 4.24	45.27 ± 2.38	**.008** ^b^
ROI-3	43.18 ± 4.21	45.45 ± 1.77	**.007** ^b^
**Tb.Th**			
ROI-1	4.09 ± 0.49	4.03 ± 0.25	.504
ROI-2	4.01 ± 0.51	3.99 ± 0.32	.891
ROI-3	4.07 ± 0.51	4.06 ± 0.23	.938
**Tb.Sp**			
ROI-1	5.05 ± 0.88	4.61 ± 0.53	**.018** ^b^
ROI-2	4.99 ± 1.04	4.62 ± 0.52	.075
ROI-3	4.92 ± 0.81	4.58 ± 0.37	**.035** ^b^
**Tb.N**			
ROI-1	1.75 ± 0.27	1.81 ± 0.12	.245
ROI-2	1.69 ± 0.26	1.78 ± 0.17	.101
ROI-3	1.74 ± 0.31	1.81 ± 0.1	.208

Abbreviations: BA/TA = bone area fraction, CBCT = cone-beam CT, FD_ct_ = cortical fractal dimension, FD_tb_ = trabecular fractal dimension, L_c_ = cortical lacunarity, L_tb_ = trabecular lacunarity, MCT = mandibular cortical thickness, MGV = mean gray value, MRONJ = medication-related osteonecrosis of the jaws, PR = panoramic radiography, Tb.N = trabecular number, Tb.Sp = trabecular separation, Tb.Th = trabecular thickness.

Bold values indicate statistically significant.

a Independent samples *t*-test.

b *P* < .05.

MGV and BA/TA values of the MRONJ group measured in PR were lower than the measurements of the control group on ROI-1 (*P*_mgv_ = .004, *P*_ba/ta_ = .004), ROI-2 (*P*_mgv_ = .007, *P*_ba/ta_ = .008), and ROI-3 (*P*_mgv_ = .008, *P*_ba/ta_ = .007). No significant differences were obtained between the MRONJ and control groups for Tb.Th and Tb.N measurements of different ROIs in PR images (*P* > .05) (Table 3).

Comparison of the measurements of different ROIs in CBCT images disclosed that while L_tb_ was higher for MRONJ-affected trabecular bone on ROI-3 (*P* = 0.014), L_ct_ was higher for MRONJ-affected cortical bone on ROI-5 (*P* = .038). However, none of the other measurements (FD, MGV, BA/TA, Tb.Th, Tb.Sp, Tb.N) revealed any difference between MRONJ-affected and healthy bone for different ROIs in CBCT images (*P* > .05) (Table 4).

**Table 4.** Measurements of different ROIs in CBCT for the MRONJ and control groups.

Parameter	MRONJ Mean ± SD	Control Mean ± SD	*P-*value^a^
**FD_tb_**			
ROI-1	1.47 ± 0.06	1.49 ± 0.03	.079
ROI-2	1.36 ± 0.05	1.36 ± 0.04	.163
ROI-3	1.37 ± 0.04	1.37 ± 0.04	.601
**FD_ct_**			
ROI-4	1.4 ± 003	1.39 ± 0.04	.445
ROI-5	1.41 ± 0.03	1.42 ± 0.03	.138
**L_tb_**			
ROI-1	0.34 ± 0.05	0.32 ± 0.03	.192
ROI-2	0.4 ± 0.07	0.4 ± 0.05	.862
ROI-3	0.36 ± 0.06	0.33 ± 0.03	**.014** ^b^
**L_ct_**			
ROI-4	0.34 ± 0.05	0.34 ± 0.05	.83
ROI-5	0.35 ± 0.04	0.33 ± 0.04	**.038** ^b^
**MGV**			
ROI-1	107.66 ± 7.62	109.84 ± 6.64	.217
ROI-2	105.34 ± 22.62	106.59 ± 8.74	.766
ROI-3	108.05 ± 9.47	104.18 ± 8.23	.08
**BA/TA**			
ROI-1	42.22 ± 2.99	43.08 ± 2.61	.219
ROI-2	42.54 ± 4.71	41.80 ± 3.42	.464
ROI-3	42.23 ± 4.10	41.04 ± 3.25	.193
**Tb.Th**			
ROI-1	5.38 ± 0.68	5.12 ± 0.61	.106
ROI-2	5.05 ± 0.71	4.88 ± 0.64	.311
ROI-3	4.86 ± 0.48	4.71 ± 0.49	.200
**Tb.Sp**			
ROI-1	6.83 ± 1.21	6.35 ± 0.77	.063
ROI-2	6.43 ± 1.1	6.46 ± 0.83	.901
ROI-3	6.04 ± 0.75	6.01 ± 0.65	.854
**Tb.N**			
ROI-1	2.24 ± 0.31	2.18 ± 0.33	.513
ROI-2	2.13 ± 0.45	2.02 ± 0.38	.27
ROI-3	2.03 ± 0.32	1.91 ± 0.31	.117

Abbreviations: BA/TA = bone area fraction, CBCT = cone-beam CT, FD_ct_ = cortical fractal dimension, FD_tb_ = trabecular fractal dimension, L_c_ = cortical lacunarity, L_tb_ = trabecular lacunarity, MCT = mandibular cortical thickness, MGV = mean gray value, MRONJ = medication-related osteonecrosis of the jaws, PR = panoramic radiography, Tb.N = trabecular number, Tb.Sp = trabecular separation, Tb.Th = trabecular thickness.

Bold values indicate statistically significant.

a Independent samples *t*-test.

b *P* < .05.

### Intra-observer agreement

The Cronbach alpha coefficients of repeated measurements of PR and CBCT images were higher than 0.9 for all measurements of both the groups (Table 5).

**Table 5.** The Cronbach alpha coefficients of included parameters.

	Control group Cronbach alpha		MRONJ group Cronbach alpha	
Parameter	PR	CBCT	PR	CBCT
MCT	0.997	0.998	0.994	1.000
FD	0.947	0.967	0.951	0.961
Lacunarity	0.933	0.956	0.935	0.961
BA/TA	0.986	0.996	0.986	0.991
MGV	0.987	0.996	0.994	0.995
Tb.Th	0.971	0.987	0.961	0.981
Tb.Sp	0.972	0.981	0.992	0.984
Tb.N	0.975	0.988	0.984	0.992

Abbreviations: BA/TA = bone area fraction, CBCT = cone-beam CT, FD = fractal dimension, L = lacunarity, MCT = mandibular cortical thickness, MGV = mean gray value, MRONJ = medication-related osteonecrosis of the jaws, PR = panoramic radiography, Tb.N = trabecular number, Tb.Sp = trabecular separation, Tb.Th = trabecular thickness.

## Discussion

Measurement of quantitative bone parameters is an objective method that can provide sufficient information about structural bone changes in early stages of the diseases causing deterioration in bone. Despite the well-proven advantages of quantitative bone measurements, MRONJ-associated trabecular and cortical bone changes have been evaluated using only a limited number of parameters. In fact, no study has previously included the evaluation of both trabecular and cortical bone measurements of MRONJ-affected bone. Moreover, there is no accepted protocol for the selection of quantitative measurement parameters specific to the radiographic imaging method(s) regarding the discrimination of healthy and MRONJ-affected bone. Therefore, the present study is first in which MRONJ-associated trabecular and cortical bone changes were comprehensively evaluated using quantitative radiomorphometric parameters in PR and CBCT images.

MCT is the most preferred quantitative parameter to evaluate the changes in MRONJ-affected bone, and its reliability has been proven by numerous studies.^5^^,^^8^^,^^18–20^ According to our results, MCT showed a significant increase in the MRONJ group both in PR and CBCT images, which is consistent with previous results. Moreover, in the current study, MCT was the only quantitative parameter that could distinguish healthy and MRONJ-affected bone in both imaging modalities, once again demonstrating the reliability of MCT in the evaluation of MRONJ-associated cortical bone changes. However, it should be noted that only patients using anti-resorptive drugs were included to this study. Even though most of MRONJ lesions occur as a result of ART,^2^ more studies with other drug types are needed to generalize our findings to all MRONJ patients.

Lacunarity is a measure of heterogeneity evaluating the dimensional distribution of gaps of the complex tissues and is used to differentiate fractal sets with completely different structures but the same FD.^28^ Therefore, the use of both FD and lacunarity has been recommended for the analysis of bone. Even though the reliability of fractal analysis in detecting osteoporosis has been proven by numerous studies,^9^^,^^11^^,^^31^ relatively few studies have used fractal analysis for the evaluation of MRONJ-affected bone.^20–23^^,^^32^ Accordingly, this is the first study including both lacunarity and FD measurements of trabecular and cortical bone of MRONJ patients. In line with previous results,^23^^,^^32^ we found that FD decreased for MRONJ-affected bone while lacunarity increased in both PR and CBCT images. Interestingly, both lacunarity and FD of trabecular bone measured only in PR images revealed a significant difference as regards the discrimination of healthy and MRONJ-affected bone. This finding indicates that FD and lacunarity measurements in PR can be reliably used to differentiate MRONJ-affected trabecular bone. Subsequently, it is possible to suggest that the heterogeneity and the dimensional variation of gaps increase as the complexity of MRONJ-affected trabecular bone decreases. Even though this was consistent with the findings of a previous study evaluating the effect of occlusal forces on the dentate and edentulous jaws demonstrating a negative correlation between FD and lacunarity,^28^ more studies with MRONJ patients are required to support our results. However, the fact remains that, PR can be safely and reliably used for FD measurements of MRONJ patients since FD is a relatively insensitive method to variations in image density, projection geometry, and ROI placement for all digital non-standardized clinical images including PR.^33–35^

The SEDENTEXCT report and many other studies strongly suggest that CBCT is not reliable for measuring bone density.^36–38^ Therefore, ratio of the sum of gray values of each pixel and total number of pixels were preferred as the method for density measurements in both PR and CBCT.^29^ The MGV of MRONJ-affected trabecular bone was significantly lower than that of healthy bone in PR images, while no difference was found in CBCT. This was contradictory to the previous CBCT studies reporting decreased trabecular density in MRONJ patients.^7^^,^^24^ However, this was not surprising because it has been already proved that the type of CBCT device, exposure parameters, FOV, and voxel size and accordingly the amount of scatter all play a key role in density measurements.^37^^,^^38^

Authors strongly recommend the combined use of morphometric parameters and bone density for the evaluation of micro-architectural bone changes.^7^ It is noteworthy that the changes in trabecular bone micro-architecture of MRONJ-affected bone have been evaluated using only micro-CT.^39–42^ Even though BA/TA and Tb.Th measurements using CBCT and periapical images were proved to be reliable in osteoporosis patients,^11^ no study have previously tested these morphometric parameters for the discrimination of micro-architectural changes of MRONJ-affected bone.

In the present study, while the increase in BA/TA of CBCT images was not significant, a significant decrease was detected for MRONJ-affected bone in PR. PR results were inconsistent with the results of previous micro-CT studies reporting increased bone volume fraction due to the ossification of bone caused by anti-resorptive drugs.^40^^,^^42^ Nevertheless, previous studies have used a single ROI positioned either on the sequestrum or on the vital sclerotic bone. However, in our study, the cumulative mean of three different trabecular ROIs was obtained in both PR and CBCT images. Thus, BA/TA of three different ROIs with variable osteosclerotic and osteolytic characteristics were measured, which was thought to be the major factor for the variability of the results. This assumption was in accordance with the findings of Wang et al^43^ indicating a variable osteoblastic and osteoclastic activity in different parts of the bone during the development of osteonecrosis. We believe that same reason applies to the inverse relationship found between BA/TA and all the other trabecular parameters as well.

Overall evaluation of all findings of included parameters reveals that the structure of trabecular bone weakens due to the decreased trabecular bone volume and density while the thickness of MRONJ-affected cortical bone increases. However, it should be remembered that osteoblastic and osteoclastic activity may vary during the development of osteonecrosis and therefore different results may be obtained for MRONJ-affected bone of patients at different stages of the disease. Due to the retrospective nature of the current study, no information can be obtained about the disease stage of the patients, therefore; further clinical studies are required to support our findings and to demonstrate the structural bone changes in different stages of MRONJ.

According to the results, PR was able to significantly distinguish healthy and MRONJ-affected trabecular bone in almost all quantitative parameters. Furthermore, most of the PR measurements plus CBCT lacunarity measurements distal to the mental foramen and above the inferior alveolar canal (ROI-3) showed significant diagnostic reliability than all the other ROIs. This finding reveals the importance of the ROI for consistency of the measurements of MRONJ-affected bone. Consequently, since the region distal to the mental foramen above the inferior alveolar canal is the only correspondent ROI for both imaging methods it can be recommended as the most reliable anatomical region for measurements of MRONJ-associated trabecular bone alterations. A similar result was reported previously for PR and CBCT fractal dimension measurements of MRONJ patients.^21^^,^^22^

As already mentioned, PR measurements showed a significant difference for almost all parameters, while CBCT measurements depicted significance only for MCT. However, it is surprising to see that the measurement values and measurement differences of PR and CBCT are very similar for both the groups (Table 2). Nevertheless, due to the higher standard deviation of CBCT measurements, no significant differences could be revealed between the MRONJ and control groups. It is well proven that image noise is the main responsible factor for the deviation of CBCT measurements particularly in images with small voxel size and high scatter.^44^^,^^45^ Since high-resolution images were used in the present study, we assume that high standard deviations caused by high image noise have played an evident role for the insignificant differences between healthy and MRONJ-affected bone in CBCT measurements. It is accepted that the distance of ROI(s) to the centre of the FOV also affects CBCT measurements as well as the location of the ROI(s) within the selected bone region.^43^^,^^46^ Due to the low number of MRONJ patients having both PR and CBCT images in our image archive, two different FOV sizes had to be used for CBCT measurements. Accordingly, the distances between the selected ROIs and the FOV centres as well as the location of the ROIs within the lesion were not standard. Respectively, these two factors may be regarded as the reasons for higher deviance of CBCT measurements. Further studies may focus on the effects of different CBCT parameters (FOV and voxel size) on the reliability of measurements of MRONJ-affected bone.

One of the limitations of this study was the small sample size of the MRONJ group due to the limited number of patients having both PR and CBCT images. Moreover, due to the retrospective nature of the study the disease stage of the patients as well as the dose of the drugs and local factors related to the development of MRONJ could not be identified since this information was not available in the patient records. Despite these shortcomings, most of the quantitative morphometric parameters measured in PR images discriminated changes in MRONJ-affected trabecular and cortical bone, confirming the clinical relevance of this study. Accordingly, we suggest that PR, which is widely used in dental clinics with its relatively low radiation dose compared to CBCT, can be reliably used for the discrimination of MRONJ-affected bone. However, further clinical studies are needed to support this presumption.

## Conclusions

According to our findings, MCT was the most reliable quantitative parameter regarding the detection of affected bone in both PR and CBCT images of patients using anti-resorptive drugs. More studies with other types of medications are needed to generalize this finding to all MRONJ patients and to recommend MCT as a primary auxiliary diagnostic parameter revealing bone changes caused by osteonecrosis process.

PR was able to significantly distinguish healthy and MRONJ-affected trabecular bone in almost all quantitative morphometric parameters. Furthermore, bone region distal to the mental foramen and superior to the inferior alveolar canal should be preferred for more reliable measurements.
